# Advances in methods for atomic resolution macromolecular structure determination

**DOI:** 10.12688/f1000research.25097.1

**Published:** 2020-07-02

**Authors:** Michael C. Thompson, Todd O. Yeates, Jose A. Rodriguez

**Affiliations:** 1 Department of Chemistry and Chemical Biology, University of California, Merced, CA, USA; 2 Department of Chemistry and Biochemistry, University of California Los Angeles, Los Angeles, CA, USA; 3 UCLA-DOE Institute for Genomics and Proteomics, Los Angeles, CA, USA

**Keywords:** Structural biology, x-ray crystallography, electron microscopy, electron diffraction, computational methods

## Abstract

Recent technical advances have dramatically increased the power and scope of structural biology. New developments in high-resolution cryo-electron microscopy, serial X-ray crystallography, and electron diffraction have been especially transformative. Here we highlight some of the latest advances and current challenges at the frontiers of atomic resolution methods for elucidating the structures and dynamical properties of macromolecules and their complexes.

## Introduction

Atomic-level structural information brings deep insight into macromolecular mechanism and function, but successful applications of structural biology methods are often challenging. Driven by the powerful insights they deliver, all of the major approaches—X-ray crystallography, multidimensional nuclear magnetic resonance (NMR), and electron microscopy—have evolved tremendously from their origins to their present forms through diverse technical innovations: improvements in instrumentation, analysis software, robotic automation, molecular engineering strategies, and so on. Major advances in structural biology have often come in waves, and structural biologists are now witnessing a sea change in the range and power of available methods for structure determination. These methodological advances are making it possible to illuminate molecular systems of growing complexity, and with sizes larger and smaller than have been possible before, at finer levels of spatial resolution. Likewise, new opportunities abound for dissecting the kinetic behavior and energetic landscapes of dynamic and polymorphic structures. Here we highlight some of the most recent developments and future prospects for applications of X-ray and electron-based crystallography and imaging methods.

## Developments in single-particle cryo-electron microscopy

For some decades following proof-of-concept experiments in cryo-electron microscopy (cryo-EM), the technique occupied a somewhat limited niche in structural biology. Single-particle imaging in cryo-EM was primarily useful for the study of large complexes, typically reaching resolutions in the nanometer range, and particularly well-suited for the study of highly symmetrical structures such as viruses, for example. The prospects for single-particle cryo-EM changed with the introduction of new detectors, microscopes, and data analysis platforms ^1, 2^. These upgrades led to an explosion in the successful use of single-particle cryo-EM methods to illuminate the detailed structures of macromolecules ^3, 4^. Cryo-EM modalities now include single-particle methods, tomography, 2D crystallography, and microcrystal electron-diffraction (MicroED). The first two modalities rely on real space imaging of either many identical copies of a molecule (single particle) or a single sample from different angles (tomography). As a diffraction method, two-dimensional crystallography has traditionally achieved the highest resolutions from highly ordered single or multi-layer protein assemblies ^5^. Based on diffraction from highly ordered three-dimensional biomolecular assemblies, MicroED has extended the attainable resolution in cryo-EM to the sub-ångstrom (Å) range using approaches borrowed from macromolecular crystallography.

The hardware and software improvements that drove the cryo-EM “resolution revolution” have enabled the technique, in favorable cases, to reach levels of detail that rival X-ray crystallography. Correspondingly, interest in cryo-EM has grown and with it a pressing need to expand accessibility to the technique. This has proven to be a major challenge owing to the high cost of purchasing and operating high-end electron microscopes. With current efforts to establish scientific centers and to develop tools that consolidate and streamline data collection, access is growing. Simultaneous efforts to improve the resolution obtainable using less-expensive microscopes are democratizing access ^6, 7^. As a result, two models are emerging for cryo-EM data collection. The first is driven by large national facilities that house high-end equipment and can be accessed by outside users through proposal-based systems. The second is an assortment of screening and data collection instruments operated independently by individual laboratories or research institutions.

### Achievements in throughput and resolution

While computational methods had been under development since the 1970s for analyzing cryo-EM images with low signal-to-noise and performing three-dimensional image reconstructions ^8^, more recent hardware and software developments opened the door for high-resolution cryo-EM ^1, 2^. Specifically, the creation and application of cameras that could detect electrons directly allowed for the collection of microscopic data as movies rather than as individual frames ^9^. This technological breakthrough led to the key data processing innovation known as “motion correction”, whereby the radiation-induced drift of particles during electron exposure could be partially corrected ^10^. With images less affected by blurring, microscopists could thereafter produce three-dimensional reconstructions of macromolecules at near-atomic resolution.

**Figure 1.**
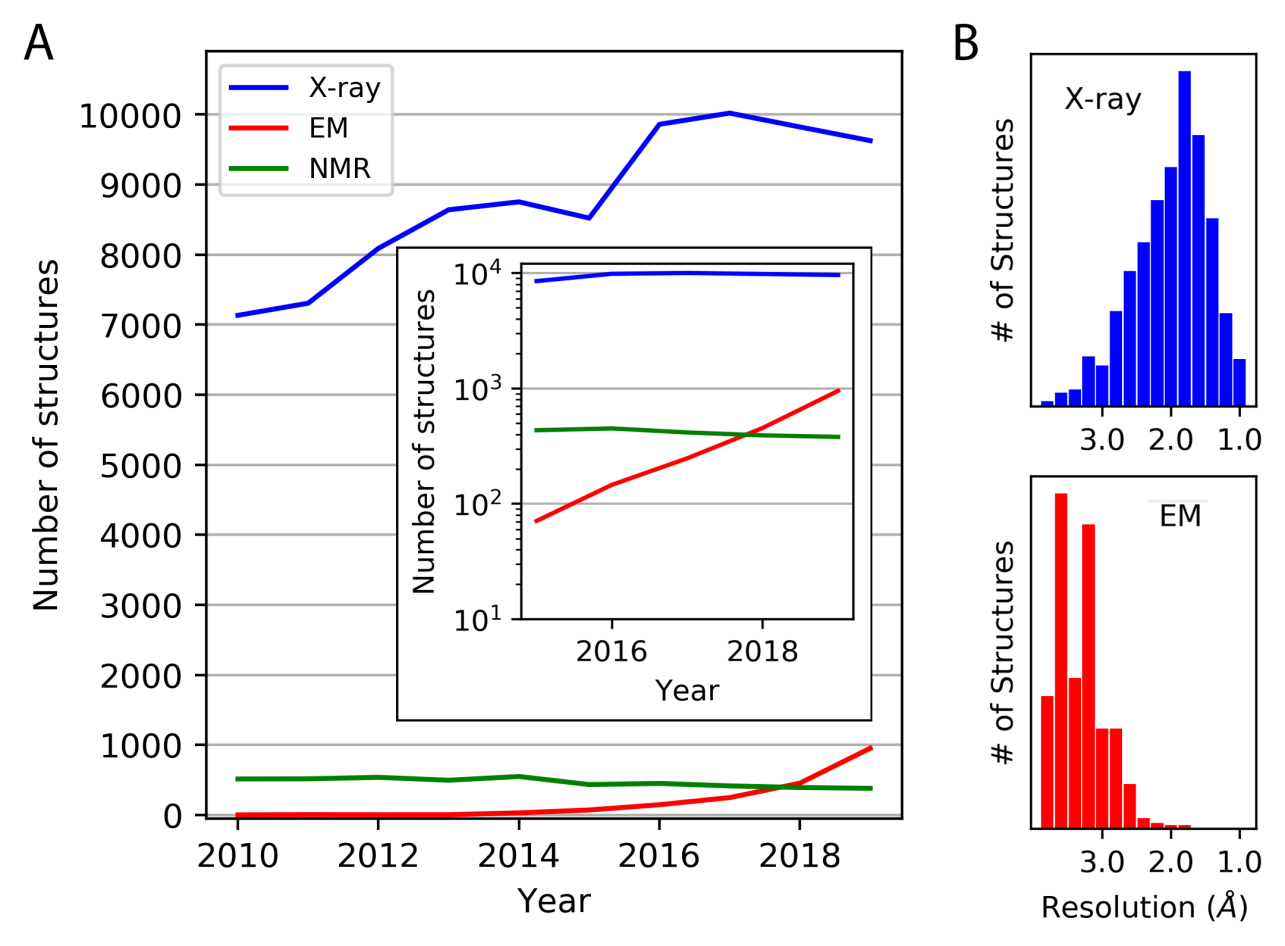
Deposition of atomic or near-atomic-resolution structures in the Protein Data Bank according to different experimental methods. ( **A**) The number of structures deposited annually is shown between 2010 and the end of 2019, based on X-ray diffraction, cryo-electron microscopy (EM), and NMR methods. A sub-4 Å resolution criterion was imposed (for X-ray and EM). The inset shows the depositions since 2015 on a logarithmic scale. ( **B**) The same set of structures are broken down by resolution. Those based on EM do not include those determined by electron diffraction methods (see Figure 4). This figure is an original image created by the authors for this publication.

**Figure 2.**
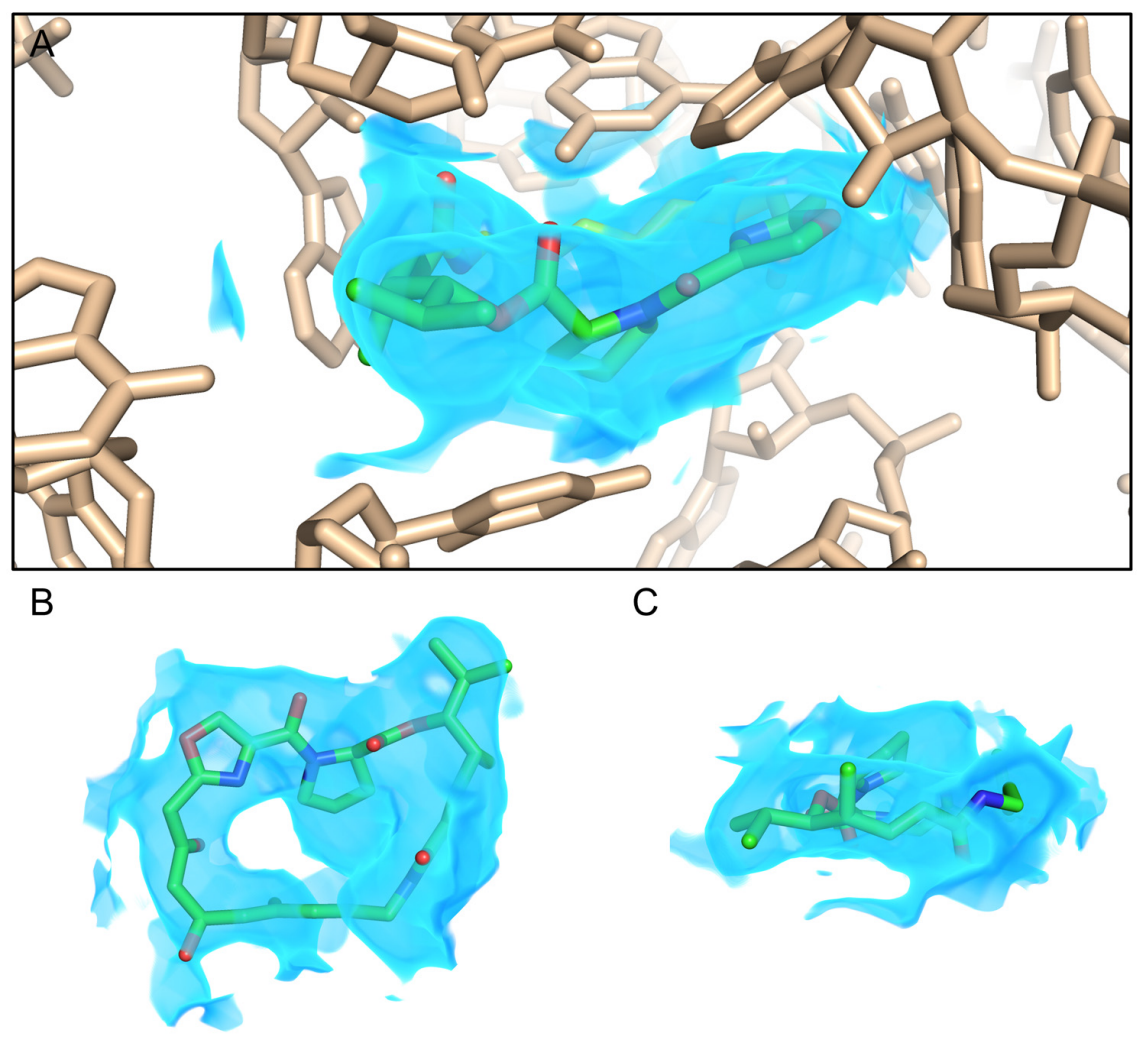
High-resolution cryo-electron microscopy (EM) as a tool for drug discovery. Cryo-EM density at a resolution of approximately 2.6 Å reveals a streptogramin antibiotic (green sticks) bound to the peptidyl transferase center (PTC) of a bacterial ribosome (wheat sticks), as described by Li, Pellegrino, *et al.* ( https://doi.org/10.26434/chemrxiv.8346107.v2). The map is shown as a blue volume and is rendered only within 2 Å of the antibiotic for clarity ( **A**). Views of the streptogramin molecule (ribosome deleted) normal to ( **B**) and coplanar with ( **C**) the macrocycle ring illustrate features such as the proline in the macrocycle backbone, isopropyl side chain, and carbonyl groups, allowing unambiguous placement of the drug in order to inform structure-based design. This figure is an original image created by the authors for this publication.

The dramatic improvements in image quality fueled a flurry of software developments directed toward automating data collection and processing and producing more accurate three-dimensional image reconstructions ^11– 13^. The current explosion of cryo-EM technology has also uniquely benefitted from coincident advances in computer science. Cryo-EM software developers have capitalized on high-performance GPU computing ^14– 16^, cloud computing ^17, 18^, and machine learning approaches for nearly user-free data processing ^19– 21^. The resulting increase in throughput is evident ( Figure 1). To date, there are approximately 3,000 density map depositions and 2,000 atomic coordinate set depositions by cryo-EM at better than 4 Å resolution. If the exponential growth were to continue—we estimate the doubling time over the last 5 years to be about 1.1 years—then structure depositions by cryo-EM would be similar in number to those by X-ray diffraction by about 2023. The improvements in resolution are also notable. An example near the current resolution limit is a 1.65 Å structure of apoferritin ^13^. Other recent reports in the literature, including structures of ribosomes bound to antibiotics ^22^ and a TRP channel bound to capsaicin and other vanilloid ligands ^23^, highlight the ability of cryo-EM to achieve the resolution needed for structural biology applications in areas such as drug discovery ^24^ ( Figure 2).

### Advances in imaging difficult macromolecules

Large macromolecular assemblies generally provide the easiest targets for cryo-EM, owing to the high signal-to-noise obtained in individual particle images. Recent successes elucidating the structures of large macromolecular complexes are too numerous to list here. Other categories of macromolecules are coming under increasing attention. Methods for analyzing filamentous assemblies, developed over the years in studies of filamentous viruses ^25– 27^ and microtubules ^28, 29^, are being applied to an increasing variety of systems ^30– 34^. Of particular interest in medicine, amyloid proteins and polypeptides, which assemble in ways that tend to be incommensurate with three-dimensional crystal formation, have been analyzed recently by cryo-EM, with numerous studies reaching near-atomic resolution ^35– 42^.

Membrane proteins, which have been categorically difficult to crystallize and analyze by crystallography, have been challenging to study by cryo-EM as well. Nanodiscs—lipid bilayer disks bound by encircling protein molecules ^43^—are providing new and fruitful routes to analyzing membrane proteins by cryo-EM ^44– 47^. To date, roughly 70 membrane protein complexes have been determined by cryo-EM in nanodiscs.

Proteins (and nucleic acid molecules) with molecular weights below about 100 kDa are especially challenging targets ^48, 49^. In a few favorable cases, near-atomic resolution has been possible for protein or enzyme assemblies in the 40–70 kDa range ^50– 53^, but smaller proteins remain below practical (and perhaps theoretical) limits. Over the years, various ideas have been explored for using larger known structures—e.g. viral capsids, ribosomes, DNA arrays, and antibody fragments—as ‘scaffolds’ for attaching smaller ‘cargo’ proteins to make them amenable to cryo-EM imaging ^54– 58^. Problems, especially attachment flexibility between the scaffold and cargo, hindered much prior work. Recent advances have been made by adapting an alpha helical fusion approach, developed earlier in the area of protein design ^59, 60^, to achieve more rigid connections between components ^61– 63^. The use of a modular adaptor system based on DARPins, as introduced by Liu *et al.* ^62^, has allowed the display and cryo-EM visualization of cargo proteins bound non-covalently to scaffolds built from symmetric protein assemblies ^63, 64^. Specific loop sequence mutations in the DARPin (or other adaptor protein) required to bind a given cargo protein can be identified experimentally based on separate laboratory evolution studies (see 65 for a recent review of DAPRin applications). Sub-4 Å resolution has been achieved by a scaffolding system of this type ^57^. An important advance in visualizing RNA molecules in the 40 kDa range has also been demonstrated recently using a Volta phase plate to enhance image contrast, resulting in sub-4 Å resolution ^66^.

While recent advances in working with difficult types of proteins have been notable, key challenges remain. Many studies are limited by protein denaturation at the air–water interface, combined with the tendency of proteins to adopt preferred orientations on EM grids ^67, 68^. Improved methods of sample preparation and freezing are being investigated to mitigate these challenges ^69^.

Cryo-electron tomography (cryo-ET) offers new promise, along with unique challenges of its own. Samples, often of intact cells or tissues, are tilted under low-dose exposure, allowing the reconstruction of three-dimensional images of subcellular structures and their constituent macromolecular assemblies (reviewed in 70, 71). This has led to impressive views of macromolecular machines and the cellular milieu. Key challenges in cryo-ET concern resolution, which is limited by electron dose per volume. Important advances are facilitated by averaging; methods involving “sub-tomogram” averaging boost the signal-to-noise for particles present in large copy number or those exhibiting high internal symmetry, with concomitant improvements in resolution (reviewed in 72). Sample thickness is a further limitation, and focused ion beam (FIB) milling, which carves out a thin section of sample on the EM grid prior to electron beam exposure, is making a major impact ^73, 74^. Given resolutions typically insufficient to trace protein backbones and establish amino acid sequences, protein labeling and identification in a cellular context by cryo-ET is another area of vital investigation ^75^.

### Improvements in interpretation

The resolution revolution led to a series of challenges and opportunities related to the interpretation of single-particle cryo-EM data. First, the ability of microscopists to reconstruct density maps with near-atomic resolution required the development of methods and software ^76^ that could be used to build and refine ^77– 79^ molecular models from cryo-EM data, in some cases utilizing complex automated modeling ^80, 81^ or molecular dynamics calculations ^82– 85^. The new breakthroughs in cryo-EM resolution resulted in a new type of experimentally derived atomic model, which in turn necessitated the development of validation methods to assess data and model quality ^86, 87^. This was achieved through repurposing of existing tools for analysis of molecular geometries ^88^, which had already been used for years by X-ray crystallographers, as well as through the creation of new tools that analyze data quality ^89– 91^ and the fit of atomic models to three-dimensional density map reconstructions ^92, 93^.

An emerging frontier in cryo-EM data interpretation is the analysis of energetic landscapes and the dissection of structural heterogeneity ^94^. In a cryo-EM data set with a large number of particles, the equilibrium ensemble of conformational states should be statistically represented, offering the ability to model different conformations of a molecule or complex from a single sample if one can correctly classify the particles into groups sharing the same conformational state. A number of different computational strategies for performing this task are under active development ^58, 58, 95– 98^. With sufficiently large data sets, it should be possible to structurally and energetically characterize the conformational landscape and functional motions for complex molecules ^99– 102^. Finally, for systems where computational sorting of alternative conformational states is challenging, the field of antibody engineering has increasingly come to the rescue. For example, engineered antibody fragments of various types developed through library selection methods have been used to trap specific conformational states of interest ^103, 104^ (reviewed in 105, 106) and/or to act as fiducial markers for molecules and complexes that have ambiguous orientations, sometimes due to pseudosymmetry ^107, 108^.

## Developments in X-ray crystallography

While rapid developments in cryo-EM have placed it in the spotlight over the past several years, X-ray crystallography remains the workhorse of structural biology, yielding the majority of structures deposited annually into the Protein Data Bank (PDB) by a wide margin ( Figure 1a). Additionally, for structural studies of small soluble proteins that require Å-level precision, as is often the case in the fields of enzyme catalysis, drug discovery, and protein engineering, X-ray crystallography still reigns supreme ^109^ ( Figure 1b). X-ray crystallography has benefited from decades of research on automation. Modern synchrotron beamlines currently measure hundreds of samples per day, with very little human intervention ^80, 109– 111^. This rapid data collection has enabled experiments such as crystallographic fragment screening, which combines high-throughput compound screening with high-resolution structural measurements ^112– 115^.

Two major synergistic advances in X-ray crystallographic data collection and interpretation have driven exciting technological developments that complement more traditional experimental measurements. These developments have broadened the scope of questions that can be addressed using the method. First, the view of X-ray crystallography as a method for determining a single, static structure of a molecule is evolving, as several recent developments are opening exciting opportunities for extracting information about the conformational landscape by visualizing multiple structural states for a crystallized macromolecule. Second, the dramatic increase in photon flux at synchrotron beamlines and the unprecedented X-ray pulse energies available at XFEL sources have opened the door to measuring diffraction from very small or highly radiation-sensitive macromolecular crystals, enabling a variety of interesting new experiments. While the majority of crystallographic experiments are still performed using traditional measurement strategies, recent examples from the literature, some of which are highlighted below, demonstrate the deep mechanistic insight that can be obtained using exciting new experimental methods.

### Crystallography at near-physiological temperatures and modeling the conformational ensemble

A bane of traditional crystallography has been its mainly static nature, notwithstanding the important information contained in atomic displacement parameters and sparsely modeled alternative conformations. In the late 1970s, cryocrystallography—wherein a crystal is frozen in liquid nitrogen and maintained at cryogenic temperature (~100 K) throughout the course of data collection—took over the field of macromolecular crystallography ^116, 117^. Importantly, cryocooling increased the tolerance of crystals to radiation damage, a common limiting factor for successful data collection, by approximately two orders of magnitude ^118– 121^. While it was appreciated several decades ago that cryocooling would alter the intrinsic dynamics of a crystallized macromolecule ^122– 124^, the effect of cryocooling on the interpretation of mechanism was not well documented until 2009, when a study of proline isomerase demonstrated that a crystal structure determined at 100 K showed only a single side chain conformation for most residues, whereas a structure determined at 277 K revealed a series of alternative side chain conformations that were shown by mutagenesis to be critical for catalysis ^125^. In the decade since this key observation was made, a number of additional cases have demonstrated that structural information derived from data collected closer to physiological temperature (or across a range of temperatures) can provide a wealth of information about the conformational ensemble beyond a single static structure ^115, 126– 128^. As noted above, similar ideas are emerging in electron microscopy ^94^.

Performing crystallography at non-cryogenic temperatures is now easier than ever because modern X-ray detectors and data processing software enable the measurement of X-ray diffraction using permissibly low X-ray doses ^129– 131^. Additionally, computational modelling software that can use experimental X-ray diffraction data to create models of alternative conformations or entire conformational ensembles is now available ^132– 136^, allowing crystallographers to maximize the amount of structural and biophysical information that can be accessed from data collected at non-cryogenic temperatures. Frontiers in this area of research include the measurement of diffuse (non-Bragg) scattering for the analysis of protein dynamics ^137– 141^ and the incorporation of full molecular dynamics simulations (of crystallized molecules) into the modelling and refinement procedure ^142– 145^.

### Serial crystallography

**Figure 3.**
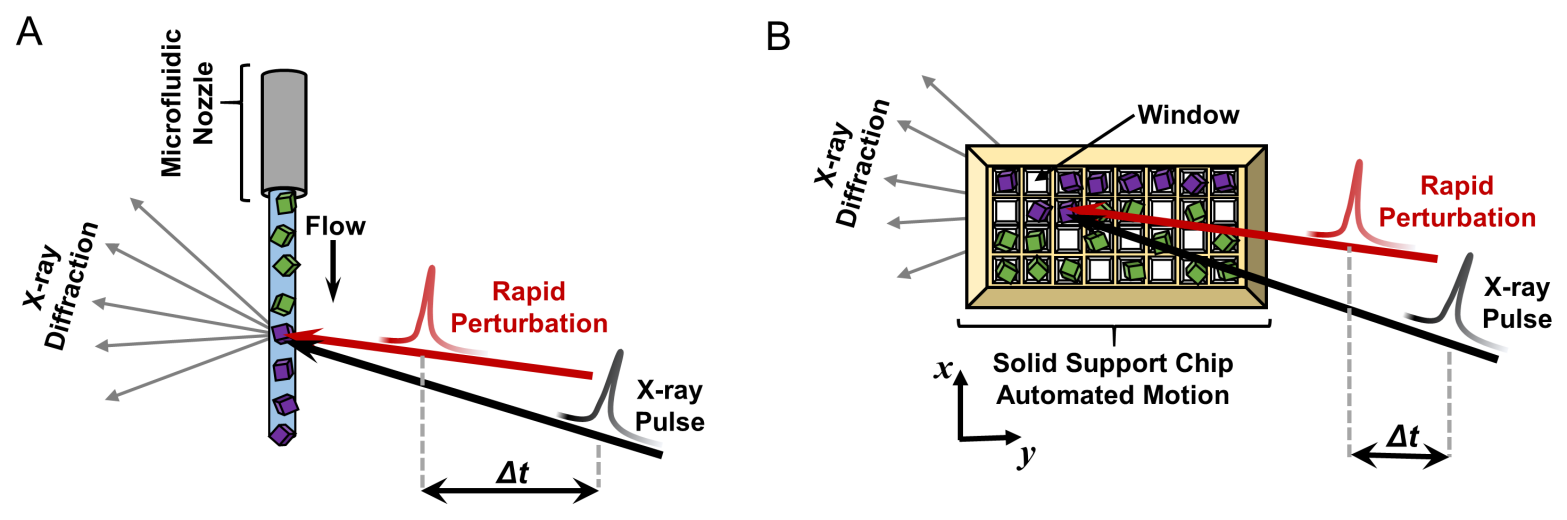
Sample delivery strategies for serial crystallography. In the microfluidic variety of the experiment ( **A**), crystals are delivered to the X-ray beam using a microfluidic nozzle ranging from tens to hundreds of microns in diameter. A stream of randomly oriented microcrystals continuously flows perpendicular to the pulsing X-ray beam (black arrow). In the fixed-target version ( **B**), microcrystals are mounted (by pipetting) in random orientations on a solid support chip that contains appropriately sized windows, and the chip is rapidly moved through the pulsing X-ray beam (black arrow) by automated translation in the x and y directions. In both diagrams, each crystal yields a single diffraction pattern capturing a random slice of reciprocal space. Crystals that have not been probed by the X-ray beam are colored green, and those that have been measured, and destroyed, are depicted in purple. In time-resolved serial crystallography, a perturbation (shown by the red arrow) is applied to the crystals with user-defined timing prior to the X-ray pulse ( *Δt*). This figure is an original image created by the authors for this publication.

Within the first decade of this millennium, first light was achieved at an X-ray free electron laser (XFEL). With it, macromolecular crystallographers gained access to a powerful tool that offered exciting new opportunities but which also required a reformulation of the traditional crystallographic experiment. XFELs produce ultrafast (tens of femtoseconds) X-ray pulses with gigawatts of peak power ^146^. The benefit of this extreme X-ray fluence is the ability to measure useful X-ray diffraction signals from extremely small macromolecular crystals, as small as several hundred nanometers ^147– 149^. A concomitant challenge, however, is that the X-ray pulses are so intense that they strip atoms of their electrons and cause Coulombic explosion of the sample ^150^. Diffraction is a nearly instantaneous process, and scattered X-rays can be recorded before destruction of the sample ^151^. However, owing to the severe damage, a single crystal cannot be used for the repeated measurements required to fully sample three-dimensional reciprocal space. This required the development of a new experimental strategy, coined “serial crystallography” ^152^. The technique was initially developed and refined prior to the operation of the first XFEL, at synchrotron facilities ^153^, and has now been successfully implemented at synchrotrons worldwide ^154, 155^. Instead of growing large, single crystals for data collection using the rotation method, serial crystallography relies on slurries of tiny microcrystals (tens of microns or less), which are serially replenished in the X-ray beam as they are destroyed by the intense X-ray pulses. Typically, a single X-ray pulse is used per individual microcrystal, destroying it in the process. Serial crystallography relies on rapid delivery of crystals to the X-ray beam, ideally in random orientations so that all reciprocal space can be sampled uniformly from thousands of microcrystals. These two needs can be met using either a microfluidic or a fixed-target approach ( Figure 3). In the microfluidic setup, a freestanding stream or jet of microcrystals is generated perpendicular to the X-ray beam, and crystals are measured as they flow through the interaction region ^156– 160^. Alternatively, in a fixed-target experiment, many microcrystals are simultaneously mounted on some type of solid support chip, which is then rapidly translated through the X-ray beam using robotics to expose the crystals for measurement ^161– 166^. Additional experimental systems that deliver microcrystals via a drop-on-demand system have also been developed, but these are not yet as widely used ^167, 168^.

The development of serial crystallography and other types of multi-crystal experiments has opened the door to interesting new opportunities for structural biology. While serial crystallography was invented to enable measurements at XFEL sources, similar experiments are now also routinely performed at very bright synchrotron beamlines ^169– 172^, making them more easily accessible to the scientific community. Because these approaches use many crystals to construct a complete data set, traditional concerns about radiation damage become unimportant, either because of the “diffraction-before-destruction” phenomenon observed at XFELs or because the required X-ray dose per crystal is much lower than for traditional experiments when performed at a synchrotron source ^173, 174^. Consequently, serial crystallography is generally performed at room temperature ^175^, the benefits of which have already been described, and the approach has proven useful for studies of radiation-sensitive samples, such as metalloproteins ^176– 179^. Furthermore, because serial crystallography enables efficient measurements from small crystals, the technique has produced crystal structures of challenging targets for which large crystals could not be obtained, including proteins such as GPCR–arrestin complexes ^180^ and RNA polymerase ^181^. Some proteins naturally form small crystals within cells, as exemplified by early structures of virus polyhedra ^182, 183^; serial X-ray methods have proven useful in structure determination for multiple types of such *in vivo* protein crystals ^184– 186^. Measurement of very small crystals with highly coherent X-rays can also produce signal between Bragg peaks. Solving the phase problem from an oversampled molecular Fourier transform represents an additional frontier in the field ^187– 191^. Finally, because serial crystallography allows the rapid measurement of many crystalline specimens, it is possible to exploit clustering methods that allow grouping of similar measurements, potentially leading to additional structural insight through the comparison of crystal polymorphs ^192, 193^.

The advent of serial crystallography using femtosecond XFEL pulses has led to a renaissance in time-resolved structural studies of macromolecules ^194^. Traditional high-resolution structural techniques are ensemble measurements, which yield information only about conformational states of molecules that are significantly populated at equilibrium. This limitation makes it challenging to study macromolecular dynamics, because protein motions can involve the formation of transient, high-energy configurations, on timescales shorter than traditional X-ray measurement. Although challenging to study, dynamics are vital for function. Dynamics play fundamental roles in enzyme catalysis ^125, 195– 198^, protein–protein interactions ^199^, allosteric signaling ^115^, and protein evolution ^127, 200^. Dynamics also have important practical implications, as they can result in the formation of cryptic binding sites that are actionable for drug discovery ^201– 203^.

In the late 1980s and early 1990s, several groups demonstrated the first successful time-resolved macromolecular X-ray crystallography (and solution scattering) experiments, essentially bringing together elements of ultrafast pump-probe spectroscopy and X-ray structural analysis to observe molecular motions ^204^. In these experiments, a rapid perturbation was applied to the crystallized proteins to synchronize conformational changes ^205^, and then one of two methods was used to observe the resulting dynamics in a time-dependent manner. In one form of the experiment, the initial perturbation was followed by freeze-trapping of the excited states by rapid cryocooling at defined time delays, followed by X-ray structure determination using traditional methods ^206– 209, 209^. Because protein dynamics are dependent on external perturbations, but also highly temperature-dependent, the freeze-trapping method was determined to be of limited utility owing to challenges in data interpretation. It was soon replaced by methods that could utilize the pulsed nature of the synchrotron beam (approximately 100 ps pulse duration) to act as a high-speed camera, capable of capturing structural snapshots of the motions in real time ^210^. By varying the time delay between the perturbation and X-ray pulses, researchers could capture molecules in various stages of conformational transitions ^211– 214^. Time-resolved crystallography (and X-ray solution scattering) proved to be an extremely powerful tool for studying macromolecular dynamics ^215– 217^, but the need to synchronize conformational changes in a significant fraction of the crystallized molecules still posed a substantial technical challenge, and time-resolved measurements were relegated to a handful of systems with well-characterized photoactivity, such as myoglobin and photoactive yellow protein, where it was straightforward to initiate conformational changes using a pulsed laser.

The use of serial crystallography for time-resolved studies has facilitated a series of recent technological developments and experimental achievements ^194^. Studies have revealed the sub-picosecond chromophore gymnastics that result in photoactivation of photoreceptors and fluorescent proteins ^218– 222^. The long-sought mechanism of photosynthetic water splitting by photosystem II has been revealed using ultrafast time-resolved crystallography paired with simultaneous X-ray spectroscopy ^223– 225^. Covalently bound enzyme–substrate reaction intermediates, which had been theorized but never observed, have been identified ^226, 227^. Additionally, a substantial effort has been made to expand time-resolved measurements to systems that do not have any photoactivity, making these experiments amenable to essentially any protein of interest. New perturbation methods that have been successfully utilized include temperature-jump ^228^, rapid mixing ^226, 227^, ligand or substrate photocaging ^229^, and rapid application of electric fields ^230^. Finally, it is worth noting that the success of time-resolved crystallography has inspired the more recent development of time-resolved cryo-EM ^231^. In such an experiment, molecules are mixed as they are sprayed onto a cryo-EM sample grid, and a time delay is introduced between the mixing and vitrification processes ^232– 234^. The time-resolution of these experiments is limited to tens of milliseconds by the time required to vitrify the sample, but they have been successfully applied to study relatively slow motions of large complexes, such as ribosomes during protein translation ^235^. Although limited in their temporal resolution, these experiments offer the opportunity to study large-amplitude motions that are incompatible with a crystal lattice.

## The resurgence of electron diffraction

For the past half century, the use of electron diffraction remained limited, often applied to unique samples or used as a complement to X-ray studies for macromolecular analysis. Common approaches to electron diffraction relied on the electron microscope, often configured to sample from the back focal plane of its objective lens. Use of the electron microscope as a tool for three-dimensional characterization of macromolecules dates to the mid-twentieth century when Klug and colleagues pioneered molecular electron microscopy at the MRC Laboratory of Molecular Biology in Cambridge ^236, 237^. In contrast to single molecules or small molecular assemblies, extended two-dimensional molecular crystals are ideally suited for diffraction. Translational invariance in the transforms of two-dimensional crystals facilitates the measurement of high-resolution diffraction. This was exploited by Henderson and Unwin to characterize crystals of bacteriorhodopsin (bR) ^238^. In fact, their use of electron diffraction necessitated thin crystals of bR to avoid extensive multiple scattering and absorption artifacts ^239^. The potent interaction of the electron beam with the single layer of molecules in two-dimensional bR crystals was sufficient to yield an initial molecular resolution structure of the protein and later several atomic models ^240, 241^. Two-dimensional electron crystallography has since produced a number of important high-resolution structures, including the plant light-harvesting complex ^242^ and aquaporin ^243– 245^. At 1.9 Å, the structure of the water pore protein, aquaporin-0, was the highest resolution structure determined by two-dimensional electron crystallography ^246^. The small quantity of high-resolution structures determined by electron crystallography may be because of its unique sample demands. For two-dimensional crystals, missing wedge effects impact resolution in the direction normal to the molecular layer, while crystal bending and in-plane defects may limit resolution along the layer ^247^. Despite these limitations, electron microscopes offer an immediate benefit for structure determination, since phases can be obtained by direct imaging of two-dimensional crystals ^248^.

In the early 1970s, electron microscopy of protein nanocrystals in liquid-filled cells demonstrated the power of using electron beams for three-dimensional crystallography ^249^. By mitigating radiation damage, cryogenic cooling of samples further facilitated the extraction of high-resolution information from three-dimensional crystals by electron diffraction ^250^. Beam attenuation measurements in these early studies demonstrated that crystals of the protein catalase—a few hundred nanometers thick—could be investigated under the electron microscope ^251^. Despite these early efforts, electron diffraction of three-dimensional macromolecular crystals was impacted by many of the same challenges that affected two-dimensional electron crystallography. Multiple scattering and absorption effects presented added concerns ^252, 253^. These limitations would ultimately delay the widespread application of electron diffraction to three-dimensional macromolecular crystals. In contrast, three-dimensional electron crystallography of small molecules and materials continued to progress, ultimately yielding structures of various inorganic and organic structures before those of macromolecules could be determined ^254– 256^.

**Figure 4.**
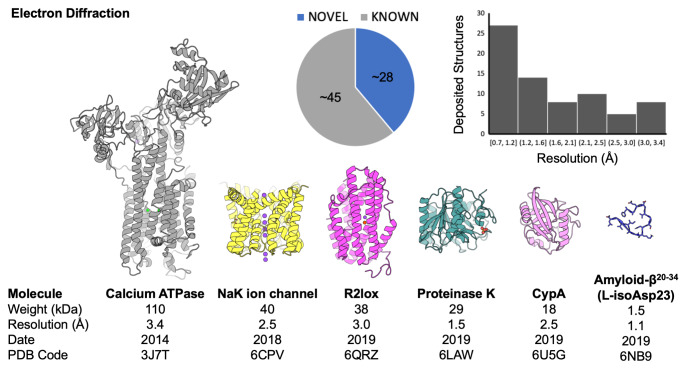
A survey of biomolecular structures determined by electron diffraction from three-dimensional crystals. An aggregate analysis of Protein Data Bank (PDB) depositions shows the breakdown of novel versus known structures and the distribution of structures according to their reported resolution (in Å). Cartoon diagrams of several representative structures are shown; below each structure are properties (molecular weight and resolution) as well as identifiers (date associated with deposition release and PDB code). This figure is an original image created by the authors for this publication.

Electron diffraction is now widely used for the study of three-dimensional inorganic and organic crystals ^257^. Its application to three-dimensional protein crystals re-emerged recently in work by Gonen ^258^, Abrahams ^259^, Yonekura ^260^, and many others. These efforts benefitted from several compelling aspects of electron diffraction, including the large electron scattering cross section, the shorter electron wavelength (and flatter Ewald sphere) in conventional electron microscopes, and the ability of the method to adopt many of the computational developments and software tools honed for X-ray crystallography over the last several decades ^261, 262^. This lowered the barriers for application of the method. However, the early studies in three-dimensional electron diffraction of protein crystals brought to light key distinctions between the use of X-rays and electrons for crystallography. Because of the strong interaction of electrons with matter, electron crystallography of three-dimensional protein crystals resuscitated concerns over multiple scattering, the resulting fidelity of diffraction measurements, and ultimately the accuracy of structures determined by these methods ^253^. While some of these concerns have been dispelled by the determination of novel and *ab initio* macromolecular structures ^263– 266^ ( Figure 4), there is considerable room for improvements in our understanding and practice of electron diffraction from protein nanocrystals.

The ability to measure diffraction from sub-micron-sized areas of three-dimensional protein nanocrystals, on the scale of the conventional domain block, has further facilitated the interrogation of individual crystallites derived from bundles or larger clusters ^267^. Crystals as thick as half a micrometer might be tolerated, but thinner specimens avoid heavy absorption and minimize multiple scattering ^268^. The term ‘MicroED’ was coined to emphasize the requirement for small crystals and is complemented by terms like three-dimensional electron diffraction (3DED) and continuous rotation electron diffraction (CRED) ^257^. Some self-associating proteins—amyloidogenic peptides, for example—tend to grow small or needle-like crystals, making them natural targets for study. For general applications, methods are being investigated for reliably producing microcrystals, or for nano-machining thin sections from larger protein crystals by FIB milling ^268– 270^. To date, less than a hundred structures determined by electron diffraction from three-dimensional crystals can be found in the PDB. Among the largest is a 110 kDa calcium ATPase; most are small proteins and peptides ( Figure 4).

One notable challenge still faced by electron diffraction is *de novo* phasing. The majority of MicroED structures deposited to date have not been novel ( Figure 4); many were determined by molecular replacement based on highly similar known structures. Some structures have been determined by direct methods, thus far only when macromolecules were small (e.g. oligopeptides) and diffracted to very high resolution. The weaker scattering for heavier atoms by electrons compared to X-rays, and the absence of anomalous scattering effects, may call for remedies to the phasing problem that could be unique to electron diffraction. For example, owing to the strength of electron scattering, information useful for structure determination might be extractable from secondary or ‘dynamic’ scattering phenomena ^252, 271^. Scattering factors for electrons are distinct from those used in X-ray experiments and may need to be further refined. Theoretical and computational efforts to more accurately analyze electron diffraction data will no doubt benefit from ongoing improvements to detectors and from the collection of energy-filtered patterns ^272^.

As electron diffraction develops beyond initial demonstrations, new opportunities and challenges arise. Recent studies have demonstrated increasingly facile structure determination of organic molecules, and the scope of substrates continues to grow. Electron nanobeams (5–100 nm in diameter) have enabled some early studies on the fine structure of macromolecular crystals and offer growth in new areas of serial or multi-structure crystallography ^273– 275^. However, the advantageous features of automation, speed, and process integration offered by most modern X-ray beamlines are not yet available for electron diffraction. Wider adoption of the method will necessarily be linked to its accessibility and its capacity to rapidly determine informative and novel structures.

## Summary

Methods for elucidating the three-dimensional structures of macromolecules continue to expand and diversify. The technical advances surveyed here push the envelope in terms of the biological systems that can be elucidated in atomic detail and the kinds of mechanistic insights that might be extracted. On one hand, improvements in robustness and automation are lowering the barriers to entry and making applications accessible to a growing body of scientists working across expansive areas of molecular biology. On the other hand, forward progress is bringing new challenges into view. Interestingly, some of the emerging challenges have historical roots, having arisen in somewhat different contexts in other areas of structural biology. The phase problem, re-emerging now in MicroED, was surmounted in the field of X-ray diffraction by heavy atom and anomalous phasing methods. The challenges of interpreting polymorphic and dynamic systems, a key goal of many of the methods discussed here, invoke connections to NMR and molecular dynamics simulation methods, which championed the study of dynamics for several decades. And efforts to expand the reach of various X-ray and electron microscopy and diffraction methods to new kinds of macromolecular systems are drawing increasingly on advances in the area of protein design. Thus, exciting developments in structural biology methods, aided by advanced computing and other approaches (including many not discussed here), are steadily expanding our ability to dissect the structure and dynamics of biological molecules in atomic detail. Combinations of different methods, new and old, should bring us closer to this ultimate goal.
